# Behavior of Lactobacilli Isolated from Fermented Slurry (*ben-saalga*) in Gnotobiotic Rats

**DOI:** 10.1371/journal.pone.0057711

**Published:** 2013-04-05

**Authors:** Williams Turpin, Christèle Humblot, Marie-Louise Noordine, Laura Wrzosek, Julie Tomas, Camille Mayeur, Claire Cherbuy, Jean-Pierre Guyot, Muriel Thomas

**Affiliations:** 1 IRD, UMR NUTRIPASS, IRD/Montpellier2/Montpellier1, Montpellier, France; 2 INRA, UMR1319 Micalis, Commensal and Probiotics-Host Interactions Laboratory, Jouy-en-Josas, France; 3 AgroParisTech, UMR Micalis, Commensal and Probiotics-Host Interactions Laboratory, Jouy-en-Josas, France; Charité, Campus Benjamin Franklin, Germany

## Abstract

Most bacterial strains, which have been studied so far for their probiotic functions, are extensively used by manufacturers in developed countries. In our work, we sought to study a mix (called BSL) comprising three strains belonging to *Lactobacillus fermentum*, *L*. *paraplantarum* and *L*. *salivarius,* that were isolated from a traditional African pearl millet based fermented slurry. Our objective was to study this BSL cocktail in gnotobiotic rats, to evaluate their survival and their behavior in the digestive tract conditions. After a single oral inoculation of germfree rats with BSL, the species established stably in the digestive tract with the following hierarchy of abundance: *L. salivarius*> *L. plantarum*> *L. fermentum*. BSL cocktail was metabolically active since it produced 50 mM lactate and it expressed genes involved in binding mechanism in the caecum. Furthermore, the global morphology of the colon epithelium was not disturbed by the BSL cocktail. BSL cocktail did not modify mucus content and host mucus-related genes (*MUC1*, *MUC2*, *MUC3* or resistin-like molecule β). The cocktail of lactobacilli enhanced the proliferating cell nuclear antigen (PCNA) at a level comparable to what was observed in conventional rats. PCNA was involved in proliferation and DNA repair, but the presence of the cocktail did not provoke proliferative events (with Ki67 as indicator), so we suppose BSL may help gut preservation. This work is the first step towards the selection of strains that are derived from traditional fermented food to formulate new probiotic mixture.

## Introduction

*Lactobacillus* has a long history of safety, and many strains have been investigated for their beneficial health effects [1]. According to its definition, a probiotic is “a live microorganism that, when administered in adequate amounts, confers a health benefit on the host” [2]. Most studies focus on a single strain such as *L. rhamnosus* GG or *L. plantarum* 299v [3], [4]. A few other studies used a mix of several bacteria such as VSL#3, which contains 8 bacterial strains belonging to *Bifidobacterium, Lactobacillus* and *Streptococcus* genus, to evaluate its beneficial effect on the host [5]. Herein we investigated the potential of a cocktail (called BSL) of three *Lactobacillus* strains: *L*. *paraplantarum* 4.4, *L*. *salivarius* 4.6, and *L*. *fermentum* 3.9.2 to induce the gut maturation of germ free rats. They were isolated from a traditional African pearl millet based fermented slurry (*ben-saalga*) and were among the dominant species of this food niche [6]–[8]. A genetic screening showed that the three strains harbor at least 21 out of the 35 genes involved in the survival within the digestive tract conditions and in the adhesion to the intestinal epithelium [8], [9]. Survival in the gastrointestinal tract (GIT) conditions is a prerequisite for the selection of probiotics and adhesion would be the base of durable health beneficial effects, such as exclusion of pathogens, immunomodulation and the increase of the duration of the beneficial bacterial molecules production such as B vitamins or bacteriocin [10]–[12]. A functional analysis showed that strains *L*. *paraplantarum* 4.4, *L*. *salivarius* 4.6, and *L*. *fermentum* 3.9.2 are able to bind to enterocytes cells HT29 and to mucus producing cells HT29-MTX, and that *L*. *paraplantarum* 4.4 was also able to express 7 out of 12 genes involved in cell binding during cell adhesion tests [9].

The bacteria present in our digestive tract are able to communicate with the host through various extracellular signals such as metabolites, growth factors, hormones, nutrients, and peptides [13]. Most studies describe the education and modulation of the immune system when triggered by the microbiota [14], [15], but the intestinal microbiota is also involved in the proliferation and maturation of the GIT [16]–[19]. Previously, we have shown that the structural maturation of the GIT by microbiota is linked to a sequential activation of different proteins involved in proliferation (Ki67 and proliferating cell nuclear antigen, PCNA), and proteins involved in cell cycle arrest protein (p21^Cip1^ and p27^Kip1^) [20]–[22]. The epithelium homeostasis of the digestive tract is essential for the prevention of injury, inflammation, protection against pathogen infection, digestion and absorption of nutrients [23], however, little is known concerning the lactobacilli-linked effect. If we consider that *Lactobacillus* are, together with bifidobacteria, pioneer bacteria colonizing a yet immature GIT [24], they may impact the maturation and homeostasis of the intestinal epithelium after birth.

The objective of this work was to study the effect of a mix of *L*. *paraplantarum* 4.4, *L*. *salivarius* 4.6, and *L*. *fermentum* 3.9.2 on the maturation of the intestinal epithelium of germ-free rats. Therefore, the ability of the three LAB to survive and establish in the digestive tract of the rats was investigated, in relation with the expression of their binding related genes. As an estimation of the host response to the presence of the bacteria, we described the mucin related gene transcripts, the production of cell cycle related proteins, as well as the colonic epithelium morphology.

## Materials and Methods

### Animals and experimental design

All procedures were carried out in accordance with European and French guidelines for the care and use of laboratory animals. Permission 78–123 is a permit number dedicated to M. Thomas. MICALIS (Microbiologie de l'Alimentation au Service de la Santé) review board specifically approved this study. The following groups of male, Fisher 344 rats were used: germ-free (GF, n = 4); conventionalized (CV, n = 4); GF inoculated with the mix of lactobacilli (BSL, n = 8) containing *L. fermentum* 3.9.2, *L. paraplantarum* 4.4 and *L. salivarius* 4.6. To obtain BSL rats, GF rats were inoculated by single oral gavage with 1 mL of inoculum containing 10^8^ CFU/mL of each strain. The CV were GF rats which were inoculated with a fecal microbiota obtained from conventional rats. CV rats harbored a microbiota and were reared in standard conditions at least for 30 days. Animals were born and bred at the Institut National de la Recherche Agronomique (Jouy-en-Josas, France). The GF and BSL rats were reared in isolators (La Calhène, Vélizy, France). All groups of rat received the same standard diet (UAR, Villemoisson, France), sterilized by gamma irradiation (45 kGy). All rats were euthanized at the age of 3 months. In the group BSL, rats were euthanized 2 or 30 days after the inoculation and were named BSL-2d (n = 4) and BSL-30d (n = 4), respectively. At 9AM, rats were anesthetized with isoflurane. The colons were removed and immediately used either for epithelial cell isolation or for histological procedure. The caecum content was frozen in liquid nitrogen and kept at −80°C until RNA extraction.

### Cell isolation procedure and protein extraction

Epithelial cells from colon were isolated according to the method described by Cherbuy et al [20], [25], [26]. Protein extraction was made on freshly isolated cells according to Cherbuy et al [20]. Briefly, the cell pellet was suspended in a lysis buffer containing 0.1% Triton X-100 and a cocktail of protease inhibitor (Roche, Mannheim, Germany). Lysis was performed for 1 h on a continuous slight agitation at 4°C. During lysis, cells were homogenized twice through a 26-gauge needle. The lysate was centrifugated (10 000×g, 4°C, 20 min), the supernatant was removed, aliquoted, and stored at −80°C until analysis. The concentration of proteins were measured according to Lowry et al [27].

### Western blot analysis

Proteins were suspended in Laemmli solution heated for 5 min at 90°C and electrophoresis was run on a 10 or 12% SDS-PAGE. After electrophoresis, proteins were transferred onto polyvinylidene difluoride membrane (Amersham Biosciences, Saclay, France). After blocking by TBS-T 1X/5% milk, membranes were incubated overnight at 4°C with the primary antibody, followed by incubation with appropriate peroxidase conjugated secondary antibodies (Jackson ImmunoResearch Laboratories, West Grove, PA). The signal was detected using the ECL + kit (Amersham Biosciences). Proteins were analyzed using anti-PCNA (GeneTex, PC-10; diluted 1/2,000), anti-p27^Kip1^ (Santa Cruz Biotechnology, sc-528; 1/500), and anti-cullin (Santa Cruz Biotechnology, sc-17775; 1/400).

### Total RNA extraction from eukaryotic cells

Total RNA was extracted from isolated colonic epithelial cells by the guanidinium thiocyanate method [28]. RNA concentration and purity were determined by absorbance measurement using a nanodrop and RNA Integrity Number (RIN) was checked with the Agilent 2100 bioanalyzer and the RNA 6000 nano labChip kit (Agilent technologies) at PICT platform (INRA, Jouy-en-Josas, France). All RNA had a RIN between 8.5 to 9.5, indicating a high RNA quality in all samples.

### Total RNA extraction from bacteria

The RNA extraction procedure was adapted from Turpin et al [9]. Briefly, 3 g of caecum content were diluted three times in 0.9% (wt/vol) NaCl solution and centrifuged twice for 10 min at 1 000×g 4°C to eliminate the caecum content and then for 10 min at 10 000×g 4°C to pellet the bacteria. The final pellet was then washed one more time in 0.9% (wt/vol) NaCl. The pellet was resuspended in 400 µl buffer (EDTA 1 mM, Tris 10 mM, pH 7, Promega) and the resulting suspension was submitted to a Tissue Lyser (Quiagen, Rheinische, Germnay) in the acid phenol pH 4 (Eurobio, Ulysse, France) and with zirconium beads (VWR, Fontenay-sous-Bois, France) to allow bacteria disruption. After centrifugation, the aqueous phase was transferred into TRIzol® (Invitrogen, Carlsbad, USA) and incubated five minutes at room temperature. After addition of chloroform (Carlo Erba, Val de Reuil, France), the solution was centrifuged (10 000×g, 15 min) and the nucleic acid was precipitated by the addition of isopropanol (Sigma, St Louis, USA). The pellet was washed with 70% ethanol (Carlo Erba, Val de Reuil, France), resuspended in nuclease free water (Promega, Madison, USA), and kept one night at −80°C. The RNA quality was check using nanodrop ND-1000 (Thermo Scientific) and bioanalyser 2100 (Agilent technologies). All RNA had a RIN between 8.0 to 9.5, indicating a high RNA quality in all samples.

### DNase treatment and Reverse transcription

The DNA was removed with RQ1 RNase-Free DNase (Promega, Charbonnières, France) and the cDNA was obtained from the Reverse Transcription System (Promega, Charbonnières, France) following manufacturer instructions. The absence of genomic DNA in treated bacterial RNA samples was checked by semi-quantitative PCR using the primers 338f, 5′-CCTACGGGAGGCAGCAG-3′ and 518r 5′-ATTACCGCGGCTGCTGG-3′ [29] specific of the 16S rRNA gene sequence of bacteria. For treated eukaryotic RNA samples, the absence of genomic DNA was checked by semi-quantitative PCR using the primers rGAPDH: 5′-TGACAACTCCCTCAAGATTGTCA-3′ and 5′-GGCATGGACTGTGGTCATGA-3′ [30].

### Semi-quantitative PCR

All experiments were performed in triplicate using the qPCR system (Stratagene, Mx3005p ™) and Syber green technology (Eurogentec, Angers, France). For each reaction, 1 µL of the cDNA template was added to 15 µL of PCR mix containing 1X Mesa green q-PCR Master Mix Plus (Eurogentec, Angers, France) and 0.3 µM of each primer (Table 1). The PCR conditions used were 10 min at 95°C and 40 cycles of 30 s at 95°C, then 30 s at 50°C or 55°C, depending on the melting temperature of primer, then 30 s at 72°C, followed by a dissociation curve from 55°C to 95°C. Absolute quantification of bacterial transcripts copy number was done by a standard curve method based on known bacterial concentration of each individual *Lactobacillus* strains.

**Table 1:** Primers used for semi-quantitative PCR.

Organisms	Genes	Name	Sequence 5′ 3′	References
*Lactobacillus*	*ef-Tu*	ef-TuF	F_ TCGATGCTGCTCCAGAAGAAA	[9]
		ef-TuR	R_ TGGCATAGGACCATCAGTTGC	[9]
	*eno*	enoF	F_ CTACCTTGGCGGATTCAACG	[9]
	*gap*	GDPH 423F	F_ ACTGAATTAGTTGCTATCTTAGAC	[76]
		GDPH 423R	R_ GAAAGTAGTACCGATAACATCAGA	[76]
	*groEl*	groElF	F_ TTCCATGGCKTCAGCRATCA	[9]
		groElR	R_ GCTAAYCCWGTTGGCATTCG	[9]
	*srtA*	srtAF	F_ ATGGGGCARGGTAACTACGC	[9]
		srtAR	R_GCCCCGGTMTYATCACAGGT	[9]
	*apf*	apfF	F_ YAGCAACACGTTCTTGGTTAGCA	[8]
		apfR	R_ GAATCTGGTGGTTCATAYWCAGC	[8]
	*cnb*	cnbF	F_ CGTGGAGAAGTCGGTGGATG	[9]
	*fpbA*	cnbR	R_ CATTGCTATGACGCCGGAAC	[9]
		fpbAF	F_ WGCYAAYCGGAAGAATCACC	[9]
		fpbAR	R_ ACCGAGTTCGTYRCGGGTCR	[9]
	*mapA*	Map 423F	F_ TGGATTCTGCTTGAGGTAAG	[76]
		Map 423R	R_ GACTAGTAATAACGCGACCG	[76]
	*mub1*	Mub 423F	F_ GTAGTTACTCAGTGACGATCAATG	[76]
		Mub 423R	R_ TAATTGTAAAGGTATAATCGGAGG	[76]
	*mub2*	mub2F	F_ ACGCGTATTGCGGGTAATGA	[9]
		mub2R	R_ CGCCCCTGAAGTGGGATAGT	[9]
	*16S rRNA for L. paraplantarum*	338r*	F_ CTGCTGCCTCCCGTAGGAGT*	[29]
		Lpla72f	R_ ATCATGATTTACATTTGAGTG	[77]
	*16S rRNA for L. fermentum*	338r*	F_ CTGCTGCCTCCCGTAGGAGT*	[29]
		Lferm72f	R_ CCTGATTGATTTTGGTCGC	[78]
	*16S rRNA for L. salivarius*	616 V	F_ AGAGTTTGATCCTGGCTCAG	[79]
		spez92R	R_ GAATGCAAGCATTCGGTGTA	[79]
	*16S rRNA for bacteria*	338f	F_ ACTCCTACGGGAGGCAGCAG	[29]
		518r	R_ ATTACCGCGGCTGCTGG	[29]
	*MUC1*	rMuc1	F_ GAGTGAATATCCTACCTACCAC	[30]
		rMuc1	R_ TTCACCAGGCTAACGTGGTGAC	[30]
	*MUC2*	rMuc2	F_ GCCAGATCCCGAAACCA	[30]
		rMuc2	R_ TATAGGAGTCTCGGCAGTCA	[30]
	*MUC3*	rMuc3	F_ AACTTCCAGCCCTCCCTAAG	[30]
		rMuc3	R_ GCTTCCAGCATCGTCTCTCT	[30]
	*TFF-3*	rTFF-3	F_ TTTGACTCCAGCATCCCA	[30]
		rTFF-3	R_ CGCAATTAGAACAGCCTTG	[30]
	*RELM-β*	rRELM-β	F_ TTCCTTCTCTCGCTGATGGT	[30]
		rRELM-β	R_ GCAGTGGCAAGTAGTTCCAT	[30]
	*GAPDH*	rGAPDH	F_ TGACAACTCCCTCAAGATTGTCA	[30]
		rGAPDH	R_ GGCATGGACTGTGGTCATGA	[30]

* The primers from the literature were converted into their reverse complement.

For eukaryote, the *GAPDH* RNA was considered as the reference gene. A set of previously designed primers were used for *MUC1*, *MUC2*, *MUC3*, *TFF-3* and resistin-like molecule β (*RELM-β*) genes expression quantification (Table 1). Results obtained were normalized to *GAPDH* RNA and compared with the mean target gene expression of CV rats as calibrator sample. The following formula was used: fold change  = 2^−ΔΔCt^, where ΔΔCt threshold cycle (Ct) equals (target Ct – reference Ct) of sample minus (target Ct – reference Ct) of the calibrator. Data were analyzed using MxPro QPCR software 2007 Stratagene version 4.10.

### Bacterial counts

Before euthanasia, the total count of bacteria in fresh feces of groups BSL-2d and BSL-30d rats was determined by plating on MRS agar after serial decimal dilutions in 0.9% (wt/vol) NaCl solution. For species determination, the transcripts of the 16S rRNA coding gene were determined in parallel for each sample using specific primers (Table 1). Absolute quantification of transcripts copy number was done by a standard curve method based on known bacterial concentration.

### Dosage of D- and L-lactate

D- and L-lactate were measured in caecal contents with the Biosentec D/L lactic acid enzymatic kits according to the manufacturer instructions (Biosentec, Toulouse, France) as described in Rul et al [21].

### Histology analysis

Colon samples were cut into 2 cm sections, fixed in 4% paraformaldehyde (4 hours, room temperature), dehydrated and embedded in paraffin. Four micrometer sections were mounted on SuperFrostH® Plus slides. Slides were stained with Hematoxylin-Eosin-Safran (HES), with alcian blue (AB) or with periodic acid Schiff (PAS) for histological analysis. Immunological staining was done with the Envision + system-horse-radish peroxidase (Dako, France) according to the recommendations of the manufacturer. Antigen retrieval was performed by boiling slides for 40 min in 0.01 mol/l sodium citrate pH 6.0. Primary antibodies used were Ki67 (clone MIB-5, Dakocytomation, dilution 1/50) and anti-PCNA (GeneTex, PC-10; dilution 1/10,000). Negative controls were performed by omitting the primary antibody from the reactions. For each section, Ki67 or PCNA-positive cells were counted on 10 crypts per rats, and results were expressed as percent of total cells per colonic crypt. Crypt depths were determined with NDP. view software (Hamamatsu). Only U shaped longitudinally cut crypts with open lumina along the crypt axis were analyzed. Results were the mean obtained by analysis of at least 10 crypts per rat.

### Presentation and analysis of data

Results are presented as means ± SEM for the number of animals indicated. Comparisons of group data were performed using one-way analysis of variance (ANOVA) followed by Dunnett's test (StatView v5.0) when the ANOVA revealed differences among the groups. Differences were considered statistically significant at P<0.05.

## Results

### Lactobacilli colonization and lactate production *in vivo*

**Figure 1:**
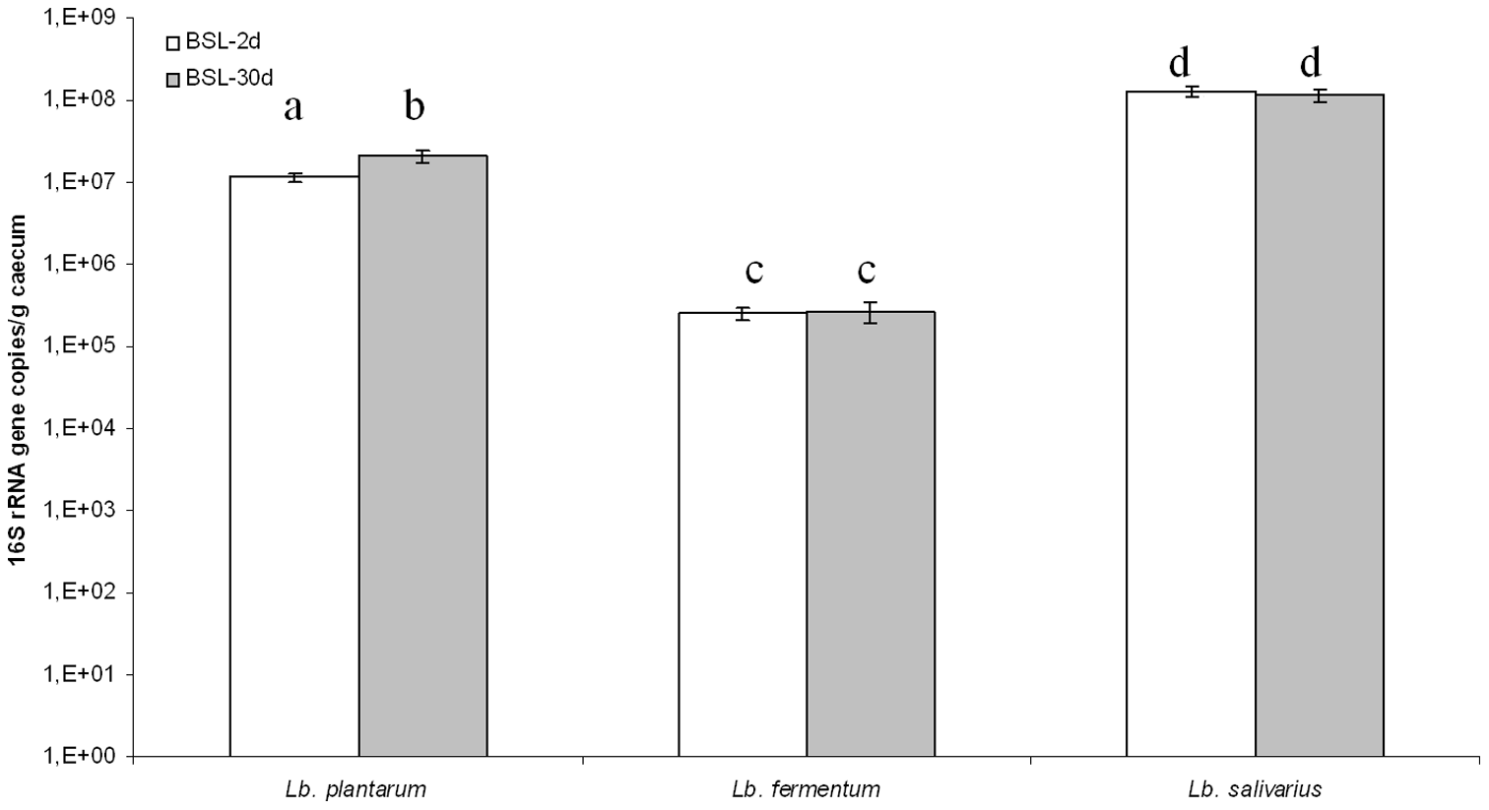
Enumeration of LAB species present two days (BSL-2d) and 30 days (BSL-30d) after the inoculation with a mix of three lactobacilli (BSL)as measured by real time PCR based on the transcripts of the 16S rRNA gene extracted from caecum of the gnotobiotic rats. Different letters indicate a statistical difference (p<0.05, Student-Newman-Keuls test). The number of 16S rRNA gene copies per gram of caecum in BSL-2d is in white. The number of 16S rRNA gene copies per gram of caecum in BSL-30d is in gray.

Total counts of bacteria in rat feces were 2.8±1.3 10^8^ CFU/g feces for BSL-2d and 3.9±0.6 10^8^ CFU/ml for BSL-30d. The three species were specifically quantified by semi quantitative PCR (Figure 1) of 16S rRNA gene transcripts extracted from the caecum. *L. salivarius* was the most abundant species in both groups of rats (1.3±0.2 10^8^ 16S rRNA gene copies/g caecum and 1.1±0.2 10^8^ 16S rRNA gene copies/g caecum for BSL-2d and BSL-30d, respectively). *L. plantarum* was ten times less represented than *L. salivarius*; there were a slight increase in its population from 2 days to 30 days after inoculation. *L. fermentum* was the least represented with 2.5±0.4 10^5^ and 2.7±0.8 10^5^ 16S rRNA gene copies/g caecum for BSL-2d and BSL-30d, respectively. The total number of each bacterium and their concentrations were not significantly different between 2 and 30 days for *L. salivarius* and *L. fermentum*. As the main metabolite of lactobacilli is lactic acid, we quantified in the caecum the L and D-lactate concentrations. BSL-2d and BSL-30d rats displayed 50.4±2.3 mM and 46.9±2.3 mM of L-lactate, respectively. D-lactate was produced at 4.4±0.2 mM and 4.2±0.6 mM in BSL-2d and BSL-30d respectively. Thus, this microbial mix constituted by three lactobacilli, was stable and metabolically active as soon as 2 days after gavage and was maintained for 30 days in the digestive tract of the animals.

### Expression of bacterial genes involved in binding mechanism

**Figure 2:**
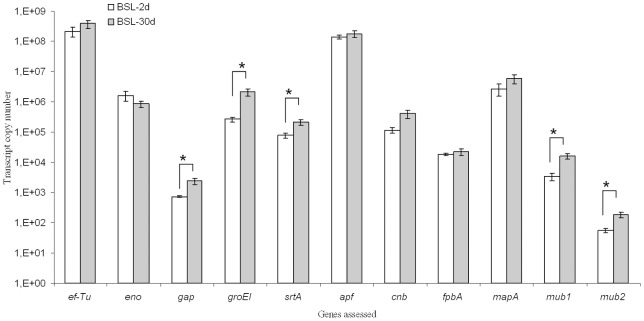
Expression of bacterial binding related genes in the bulk of LAB present in the gnotobiotic rats caecum two days (BSL-2d) and 30 days (BSL-30d) after the inoculation with a mix of three lactobacilli (BSL). Asterisk indicates a significant difference between BSL-2d and BSL-30 d groups (p<0.05, Student-Newman-Keuls test). The transcript copy number in BSL-2d is in white. The transcript copy number in BSL-30d is in gray.

In the caecum, all the bacterial genes involved in the binding mechanism (Figure S1) were expressed, the house keeping genes (*ef-Tu, eno, gap, groEl, srtA*), as well as those more specifically related to the binding function (*apf, cnb, fpbA, mapA, mub1, mub2*) (Figure 2). Most of those genes were expressed at the same level in the BSL-2d and BSL-30d rats. However, some of them (*gap, groEL, srtA, mub1* and *mub2*) were significantly more expressed in the BSL-30d group.

### Effect of the lactobacilli cocktail on mucus content and host mucus-related genes

**Figure 3:**
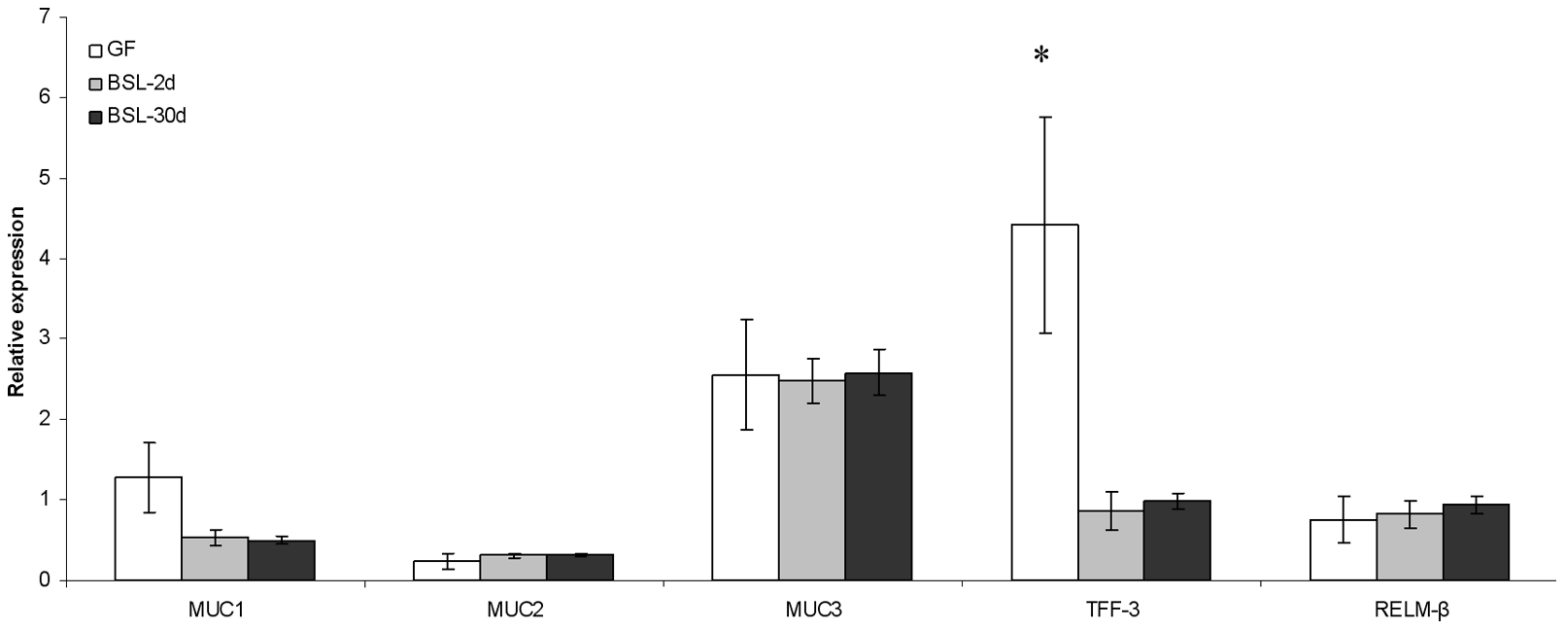
Relative expression of mucin related genes in the colon of GF, BSL-2d and BSL-30d rats compared to GAPDH expression from the conventionalized group. The relative expression of mucin related in GF is in white, in BSL-2d is in gray, and in BSL-30d is in black. Asterisk indicates a statistical difference between GF and BSL rats.

The BSL bacteria express binding related genes and we wondered if, as a response to this cocktail, there was a change in the expression of the rat mucin related genes *MUC1, MUC2, MUC3, TFF-3* and *RELM-β* in the colon of BSL-2d, BSL-30d and GF groups (Figure 3). All the calculations were made with CV rats, harboring a mature epithelium, as the reference condition. There were no differences in the expression of *MUC1*, *MUC2*, *MUC3* and *RELM-β* between GF and BSL rats. The expression of *TFF-3* was 7.4±3.1 and 4.6±1.4 fold repressed in BSL-2d and BSL-30d rats compared to GF rats. The level of *TFF-3 mRNA* was equivalent in BSL-2d, BSL-30d and CV rats.

The glycoproteins, mucopolysaccharides and glycosaminoglycans were stained by alcian blue (AB, specific of acidic mucin) and periodic acid Schiff (PAS, specific of neutral mucin) (Table 2). The percentage of AB-stained cells was similar between GF, BSL-2d, and BSL-30d. No differences in PAS-stained cells were observed between GF, BSL-2d or BSL-30d rats.

**Table 2:** Percentage of AB and PAS stained cells to total cells.

	AB stained cells (% of total cells)	PAS stained cells (% of total cells)
GF	45.6±0.2	34.9±1.8
BSL-2d	41.3±0.8	32.2±3.5
BSL-30d	43.8±1.5	31.4±3.6

### Effect of lactobacilli on the colonic epithelium proliferation parameters

**Figure 4:**
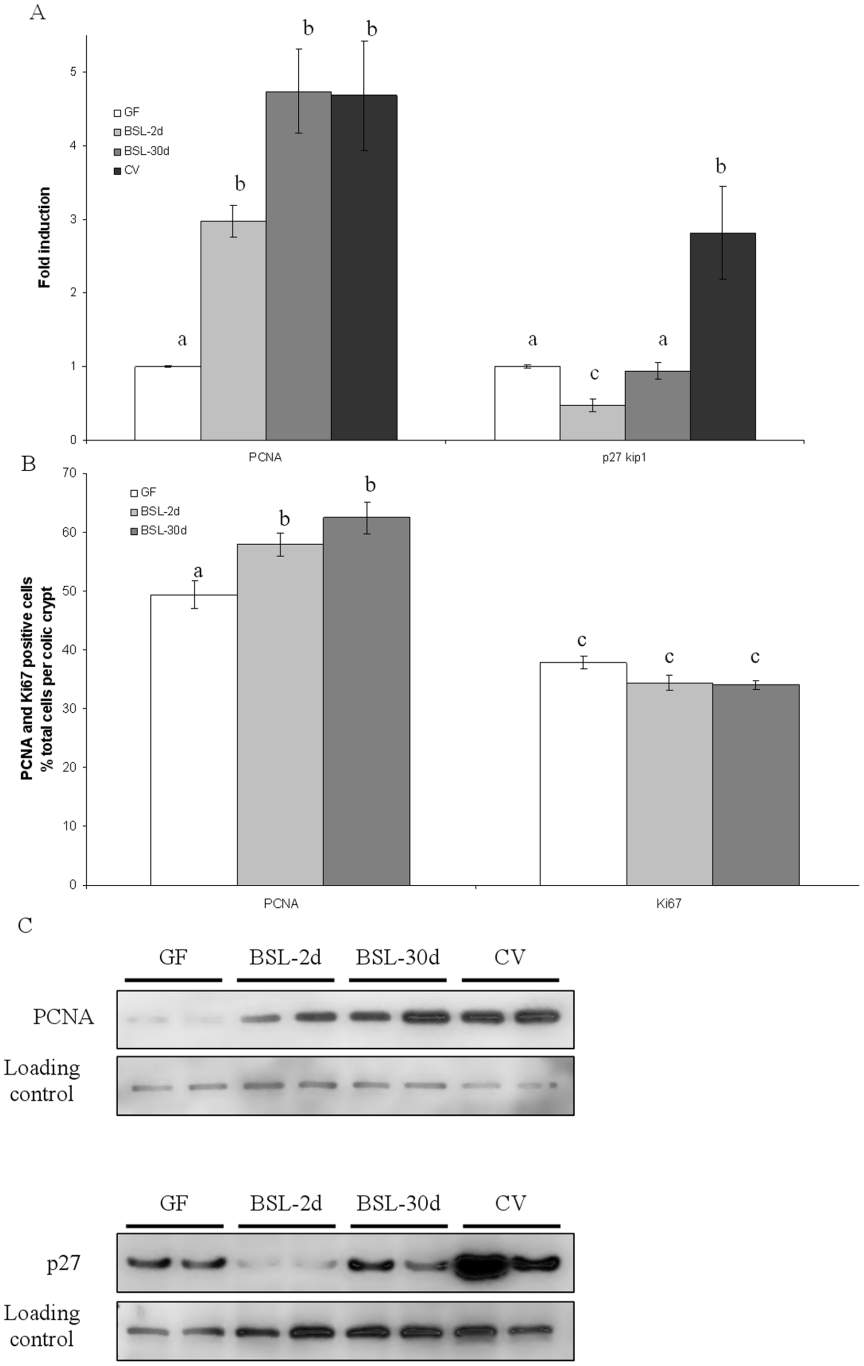
Densitometric analysis of autoradiography for amount of PCNA and p27Kip1 protein in colic epithelium in GF, BSL-2d, BSL-30d and CV rats (A) and number of PCNA and KI67 positive cells (B). Westernblot of PCNA (on the top) and p27Kip1 (on the bottom) proteins in colic epithelium in GF, BSL-2d, BSL-30d and CV rats (C). For PCNA and p27^Kip1^ quantification, cullin proteins were used as internal controls. Ki67-positive cells and PCNA- positive cells are expressed as a percentage of total colonic crypt cells. Results are presented as means ± SE for n = 4 rats per group. Statistically significant differences (P<0.05, Student-Newman-Keuls test) between groups are indicated by different letters.

The response of the epithelium to the BSL presence was also evaluated by measuring the amount of proteins known to be induced by a complex microbiota such as PCNA, which is involved in diverse functions (proliferation and repair) and p27^Kip1^, a cell cycle arrest protein (Figure 4). After inoculation with the BSL cocktail, the amount of p27^Kip1^ protein was 2.1±0.4 fold reduced in two days and became similar to the amount found in GF rats 30 days after inoculation. In contrast, PCNA amount was significantly increased in both BSL-2d and BLS-30d by 3.5±0.3 and 4.7±0.9, respectively, compared to GF rats. There was no difference in PCNA amount between BSL-2d, BSL-30d and CV rats (4.7±1.2 fold higher than in GF rats). Thus, in 30 days, the mix of lactobacilli did not change the amount p27^Kip1^ in comparison to GF, but stimulated PCNA to the level obtained with a conventional microbiota.

In regards to the change in the amount of protein involved in cell cycle regulation, we further assessed the effect of the bacterial cocktail of lactobacilli on the morphology of the colonic epithelium by immunohistochemistry. In accordance with the results obtained by western blot, the PCNA-stained cells were increased by 17.0% in BSL-2d (58.0±3.1% of total cells) and by 26.0% in BSL-30d (62.5±4.7% of total cells) compared to GF rats (49.4±3.8% of total cells) confirming western blot analysis (Figure 4). Ki67, a marker of proliferation, was also quantified by immunohistology. The percentage of Ki67-stained cells per crypt was the same among the groups (37.8±1.7%, 34.4±2.0%, 34.0±1.0% in GF, BSL-2d and BSL-30d respectively). The crypt depth of the colonic epithelium of BSL-2d (223.3±7.4 µm) and of the BSL-30d (208.6±11.9 µm) remained similar to those measured in GF rats (221.3±7.7 µm). This indicates that in comparison to GF rats, the three lactobacilli species, *L*. *fermentum*, *L*. *plantarum* and *L*. *salivarius* did not increase the absorptive surface. This was confirmed as no modulation of the molecular markers Ki67 and p27^Kip1^ was observed. However, the lactobacilli were able to induce the marker PCNA.

## Discussion

*L. paraplantarum* 4.4, *L*. *salivarius* 4.6, and *L. fermentum* 3.9.2 have been selected for their genetic equipment and their phenotypic attributes favorable to their survival in the gastrointestinal tract and their high potential for binding to the epithelial cells of the intestinal tract [8], [9]. Several studies have used a single species or a cocktail of bacteria for their probiotic potential on the host [31], [32]; however, only a few of them focused on the host response, particularly on the structural maturation of the epithelium that occurred in the presence of bacteria [20]–[22].

### BSL lactobacilli are able to set up in initially germ-free rats

The amount of BSL bacteria in the caecum was in accordance with previous studies using single *Lactobacillus* strains [33]–[35]. However, the ability of the three species to survive the passage through the gastrointestinal tract was not certain. Indeed a recent study shows that *L. bulgaricus* ATCC 11842 was not able to set up when administered alone or with *S. thermophilus* LMD9 [36]. Previously we showed that the three strains were considered tolerant to bile salts but had different survival abilities at low pH [8]. The high concentrations of the three strains in the caecum of the gnotobiotic rats, two and thirty days after a single inoculation, demonstrate their ability to survive the conditions prevailing in the proximal part of the digestive tract and to rapidly and durably set up in the large intestine. *L. fermentum* 3.9.2 was the strain with the highest survival rate at low pH [8] but was detected at a lower cell concentration level in the digestive tract. On the contrary, *L. salivarius* 4.6 did not survive at pH 2 for one hour but was found to be at a higher amount two and 30 days after inoculation. Those results would suggest that the phenotypic *in vitro* tests could have strong limitations in predicting *in vivo* survival of LAB strains and their colonization ability of the intestine. However, the condition of the *in vitro* test was rather drastic since it was performed at a very low pH, while it is now generally admitted that the gastric pH is around 4 during feeding [8], [37]. In the case of *L. fermentum* 3.9.2, it should also be considered that survival at low pH does not necessarily mean a high ability to thrive under conditions prevailing in the intestinal tract and to outcompete the other LAB.

The binding ability could also influence the implantation of these strains. Although *in vitro* studies are known to be less relevant than *in vivo* experiments, the *in vitro* binding ability of each strain measured individually on cell lines, showed that *L*. *fermentum* 3.9.2, *L*. *paraplantarum* 4.4 and *L*. *salivarius* 4.6 bound preferentially to mucus secreting cells (HT29-MTX), in comparison to non mucus secreting cells (HT29) [9]. These *in vitro* binding phenotypes were at least equivalent or higher than those of the probiotic strains *L. johnsonii* NCC 533 and *L. acidophilus* NCFM [9] and might explain their durable establishment in the caecum of rats. In addition, it is known that the use of a combination of strains instead of a single strain could enhance the overall binding of bacteria [38]. Although it is important to take into account the results obtained here, it could be different with conventional animals as the strains would need to successfully compete with endogenous bacteria.

The bacteria were alive and metabolically active in the caecum of rats as shown by agar plate counts, by the analysis of lactate production and by the transcripts measured by real time PCR. The primers for binding related genes were the same for the three *Lactobacillus* strains. So that even if they showed specific amplification of the targeted genes in each of the three *Lactobacillus* strains, as revealed by dissociations curves (data not shown), they did not distinguish between the transcripts of each strain when they are together. Therefore the transcriptional analysis corresponded to the general expression of the pool of genes investigated. Herein we showed that all housekeeping and binding related genes were expressed *in vivo*; this was similar to other results showing in gnotobiotic rodents the expression of the same genes in a large transcriptomic analysis of *L*. *plantarum* WCFS1 [39] and of *ef-Tu*, *gap* and *fpbA* in *Streptococcus thermophilus* LMD9 [21]. The *in vivo* expression of genes related to the adhesion function can be considered important for the gut colonization by LAB.

### The lactobacilli cocktail does not influence mucin genes expression and mucin amount

The colonic epithelium is covered by two layers of mucus built around the MUC2 mucin, which acts as a protective barrier against various aggressions such as bile salts, toxins, pollutants, and acts as a binding site of bacteria [40]–[43]. The expression of mucin related genes *MUC1*, *MUC2*, and *MUC3* was not induced in gnotobiotic rats harboring a caecum colonized by the LAB mix compared to GF rats. Previous studies show that the expression of *MUC1* was also not induced by *Lb. rhamnosus* GG in conventional mice, while the VSL#3 cocktail was able to induce this gene in conventional rats [31], [44]. In regard to *MUC2* gene, the presence of bacteria in cells model or in rats were able to increase its expression [9], [31], [45]–[47]. However, contrasting with these results, *L. paraplantarum* 4.4 strains were not able to induce *MUC2* expression in HT29-MTX cells, but induced expression in HT29 cells suggesting that the HT29-MTX cells are more close to *in vivo* models than HT29 cells [9]. As for *MUC3*, no modulation of the expression of the gene was obtained with *Lb. rhamnosus* GG in conventional mice [44]. However, *Lb. plantarum* 299v and the VSL#3 cocktail were shown to be able to induce *MUC3* in pathogen-free rats and in conventional rats, respectively [31], [48].

Confirming the result obtained by semi-quantitative PCR on mucin related genes, the amount of KLF4 protein, a goblet cell differentiation marker (data not shown) and the number of BA and PAS positive cells, staining acidic and neutral mucopolysaccarides respectively, remained similar in the BSL and GF rats. From these data we conclude that lactobacilli cocktail has no detectable detrimental effect on the mucus of the colonic epithelium, but has no inducer capacity on major actors involved in the mucus layer.

### Involvement of lactobacilli cocktail on cells proliferation and differentiation

The lactobacilli were not able to induce a morphological change in the colonic epithelium as indicated by the crypt depth, which remains similar two days and 30 days after inoculation compared to those observed in GF rats. Similar results were obtained with other mono associated rats [20], [22]. On a molecular basis, it is also illustrated by the similar amounts of the cell cycle arrest protein p21^Cip1^ and the proliferation marker cycline D2 between GF and BSL rats (data not shown). However, other proteins involved in the regulation of the cell cycle, and also in the maturation of the digestive tract were modulated by lactobacilli [20], [49]–[52]. This is the case of the cell cycle arrest protein p27^Kip1^, which was first reduced two days after inoculation with the BSL cocktail and then became similar to the amount found in GF rats 30 days after inoculation. In contrast, in rats inoculated for one month with *S. thermophilus* LMD9 producing 13.6±0.9 mM or LMG18311 producing 10.02±3.0 mM, p27^Kip1^ was increased by 1.8 fold and 2 fold, respectively. Similar results were obtained with intestinal line cells (HT29) incubated with 20–50 mM L-lactate, indicating that lactate could be a signal modulating the colon epithelium [21]. Herein we show that lactate was produced at concentrations above 50 mM but the p27^Kip1^ protein was not induced in BSL-30d and even repressed in BSL-2d rats. However, in the context of conventional rats, this amount is lower due to lactate-consuming bacteria. From these data on a gnotobiotic model of rat, we conclude that the regulation of p27^Kip1^ may not be only regulated by lactate and other compounds produced by lactobacilli could counterbalance the regulation of p27^Kip1^. More studies need to be addressed to identify these compounds, such as the use of killed bacteria, as some components of the Gram-positive bacterial cell wall were shown to be able to modulate host response [53].

The PCNA protein is a well-known marker to study colonic epithelial cell proliferation and it is involved in various cell processes such as cell cycle regulation, DNA replication and DNA repair [51], [54]. We show that the PCNA protein was detected at similar level in gnotobiotic rats (BSL-30d, and BSL-2d) and in CV rats. No such results were observed in rats inoculated with single strains of common inhabitants of the gut, such as: *Bacteroides thetaiotaomicron*, *Ruminococcus gnavus*, *Clostridium paraputrificum* or *S*. *thermophilus* [20], [21]. The same induction of PCNA compared to GF was observed in rats colonized by *L. plantarum* A6 or *L. fermentum* OgiE1 (data not shown). These results suggest that PCNA induction is closely related to the presence of lactobacilli rather than to the presence of several species in the same mix, *i.e.* the consortium formed by *L*. *fermentum*, *L*. *paraplantarum*, *L. salivarius*. PCNA induction was also observed *in vitro*, when *L. rhamnosus* GG and its secreted proteins, p75 and p40, were incubated with the young adult mouse colon epithelial cells [55]. The presence of a p75 homolog in our strains may explain the induction of PCNA. Indeed, we have detected by PCR a p75 homologous gene in the strains *L. paraplantarum* 4.4, and *L*. *salivarius* 4.6 (data not shown), which may be responsible for induction of PCNA in gnotobiotic rats compared to GF. Furthermore, *in silico* analysis shows that the homologous gene of p75 is not found in the genome of *S. thermophilus* LMG18311 or LMD9; both being unable to induce PCNA *in vivo*. However, the role of p40 is still unclear since none of the *Lactobacillus* strains harbor a p40 homologous gene in their genome, while *S. thermophilus* LMD9 and LMG18311 have one homolog sharing low protein identity (30.8%) with p40 of *L. rhamnosus* GG. Due to the large portion of pseudogenes in the genome of *S. thermophilus*, and the absence of PCNA induction in animals inoculated with this species, we could address the question of the functionality of p40 homologs in this species [56].

As PCNA is involved in cell cycle regulation and DNA repair, we suggest that the PCNA induction observed here may be related to an increase in the DNA repair process. Indeed, the presence of the BSL cocktail neither increased the crypt depth in the colonic epithelium or the proliferation marker Ki67. As a consequence, the induction of PCNA in gnotobiotic rats harboring the BSL cocktail is probably not linked to proliferation. Furthermore, other studies reported that *Bifidobacterium* or *L. gasseri* were also able to improve DNA repair in human cells [57], [58]. This mechanism may be associated with a tighter cross-talk with the colonic epithelium. This is suggested by the fact that *TFF-3* gene expression is decreased in BSL compared to GF rats. Another study reports that *TFF-3* is repressed in presence of *L. rhamnosus* GG in conventional mice [44]. This trefoil factor is involved in the maintenance and in the protection of the intestinal mucosal barrier, in the stimulation of epithelial cell migration and/or differentiation of epithelial cells, as well as contributes to the innate immune response [59]–[62]. We do not know if the reduced expression of *TFF-3* may be functionally linked with the induction of PCNA. Thus, further studies are required to investigate the physiological consequences of the repression of TFF-3 and the induction of PCNA triggered by the presence of BSL.

### Beneficial potential of lactobacilli cocktail

In this study we have shown that the lactobacilli cocktail is able to survive in vivo, which is a first step in the definition of probiotic [63]. Then, we have investigated if these bacteria naturally present in ben-saalga have beneficial properties in vivo as the maturation of the GIT. This is an important factor that deserves to be studied in the case of bacteria isolated from an African fermented food consumed frequently by children between 6 and 23 months [64]. In this vulnerable population, infectious episodes can alter the integrity of the intestinal mucosa and so alter the maturation of the GIT [65]. Thus, the use of LAB naturally present in ben-saalga, could improve the maturation of the GIT to help face infectious episodes [66], [67]. Herein, we have observed that the cocktail has no deleterious effect on GIT. Moreover, we have brought molecular proof demonstrating that the cocktail is able to normalize the level of PCNA protein and TFF-3 gene expression in gnotobiotic rats at the same level as in conventional rats having a fully functional gut, and so help in the maturation of the gut.

We have also demonstrated that the strains are able to produce lactate in situ. Lactate is well known to exercise antimicrobial effects and participate in the beneficial effect of *Lactobacillus* [68], [69]. In conventional animals, the lactate produced by probiotics is further fermented to acetate, propionate, or butyrate by indigenous lactate-utilizing bacteria and these products have well known beneficial activities on the gut [70], [71]. Furthermore, the lactate produced by the three strains is mainly the L-lactate form with a minor amount of D-lactate that is the deleterious enantiomer for humans [72]–[75]. As a consequence, the production of lactate by our lactobacilli cocktail may be beneficial in conventional animals or humans.

In conclusion, the three LAB species isolated from a traditional African food could potentially be candidates as probiotics, due to their colonization capacity of the intestinal tract of rats over a long period of time, which exceeds the turn-over of intestinal cells. Indeed, the establishment of LAB in the intestinal tract depends on the ability of the bacterial species to promote various factors. Results obtained here on mucin related genes are consistent with some results of the literature but also make apparent differences that prevent any sweeping generalizations since effects are strain dependent. The LAB present in the BSL cocktail were able to help in the maturation of the colonic epithelium at a molecular level by normalization of factors such as PCNA or *TFF-3* at a level similar to CV rats. From these data we can conclude that the BSL cocktail is not detrimental for the colic epithelium. This durable establishment in rats may be the initial step towards the investigation of other beneficial effects such as the *in situ* production of vitamins or immunostimulation.

## Supporting Information

Figure S1 (DOC)
