# Supplementary material for: Behavior of Lactobacilli Isolated from Fermented Slurry (ben-saalga) in Gnotobiotic Rats
Source: PLoS One. 2013 Apr 5;8(4):e57711. doi: 10.1371/journal.pone.0057711 (PMC3618507; doi:10.1371/journal.pone.0057711)
Supplement: Figure S1 — (DOC) [file pone.0057711.s001.doc]

**Figure S1: Binding related genes chosen in this study and their functional analysis**

| Gene | Predicted function | Functional analysis |
| --- | --- | --- |
| *ef-Tu* | elongation factor Tu | [1] |
|
| *eno* | enolase | [2,3] |
|
| *gap* | glyceraldehyde-3-phosphate dehydrogenase | [3,4] |
|
| *groEl* | heat shock protein 60 | [5] |
|
| *srtA* | sortase | [6,7] |
|
| *apf* | aggregation-promoting factors | [8] |
|
|
| *cnb* | collagen-binding protein | [9,10] |
|
| *fpbA* | fibronectin-binding protein | [7] |
|
|
| *mapA* | mucus adhesion promoting protein | [11] |
|
| *msa* | mannose-specific adhesin | [12] |
|
| *mub1* | mucin-binding protein | [7,13] |
|
| *mub2* | mucin-binding protein | [7,13] |
|
|
|
|  |  |  |

References

1. Granato D, Bergonzelli GE, Pridmore RD, Marvin L, Rouvet M, et al. (2004) Cell surface-associated elongation factor Tu mediates the attachment of *Lactobacillus johnsonii* NCC533 (La1) to human intestinal cells and mucins. Infect Immun **72**: 2160-2169.

2. Castaldo C, Vastano V, Siciliano RA, Candela M, Vici M, et al. (2009) Surface displaced alfa-enolase of *Lactobacillus* *plantarum* is a fibronectin binding protein. Microb Cell Fact. **16**:8-14.

3. Hurmalainen V, Edelman S, Antikainen J, Baumann M, Lahteenmaki K, et al. (2007) Extracellular proteins of *Lactobacillus* *crispatus* enhance activation of human plasminogen. Microbiology. **153**: 1112-1122.

4. Kinoshita H, Uchida H, Kawai Y, Kawasaki T, Wakahara N, et al. (2008) Cell surface *Lactobacillus* *plantarum* LA 318 glyceraldehyde-3-phosphate dehydrogenase (GAPDH) adheres to human colonic mucin. J Appl Microbiol. **104**(6): 1667-1674.

5. Izquierdo E, Horvatovich P, Marchioni E, Aoude-Werner D, Sanz Y, et al. (2009) 2-DE and MS analysis of key proteins in the adhesion of *Lactobacillus* *plantarum*, a first step toward early selection of probiotics based on bacterial biomarkers. Electrophoresis **30**: 949-956.

6. van Pijkeren JP, Canchaya C, Ryan KA, Li Y, Claesson MJ, et al. (2006) Comparative and functional analysis of sortase-dependent proteins in the predicted secretome of *Lactobacillus* *salivarius* UCC118. Appl Environ Microbiol. **72**(6): 4143-4153.

7. Buck BL, Altermann E, Svingerud T, Klaenhammer TR (2005) Functional analysis of putative adhesion factors in *Lactobacillus* *acidophilus* NCFM. Appl Environ Microbiol. **71**(12): 8344-8351.

8. Goh YJ, Klaenhammer TR (2010) Functional roles of aggregation-promoting-like factor in stress tolerance and adherence of *Lactobacillus* *acidophilus* NCFM. Appl Environ Microbiol. **76**(15): 5005-5012.

9. Aleljung P, Shen W, Rozalska B, Hellman U, Ljungh A, et al. (1994) Purification of collagen-binding proteins of *Lactobacillus* *reuteri* NCIB 11951. Curr Microbiol **28**: 231-236.

10. Hsueh HY, Yueh PY, Yu B, Zhao X, Liu JR (2010) Expression of *Lactobacillus* *reuteri* Pg4 Collagen-Binding Protein Gene in *Lactobacillus* *casei* ATCC 393 Increases Its Adhesion Ability to Caco-2 Cells. J Agric Food Chem.

11. Miyoshi Y, Okada S, Uchimura T, Satoh E (2006) A mucus adhesion promoting protein, MapA, mediates the adhesion of *Lactobacillus* *reuteri* to Caco-2 human intestinal epithelial cells. Biosci Biotechnol Biochem. **70**(7): 1622-1628.

12. Pretzer G, Snel J, Molenaar D, Wiersma A, Bron PA, et al. (2005) Biodiversity-based identification and functional characterization of the mannose-specific adhesin of *Lactobacillus* *plantarum*. J Bacteriol. **187**(17). 6128-6136.

13. Roos S, Jonsson H (2002) A high-molecular-mass cell-surface protein from *Lactobacillus* *reuteri* 1063 adheres to mucus components. Microbiology **148**: 433-442.
